# Initial clinical experience with ArcCHECK for IMRT/VMAT QA

**DOI:** 10.1120/jacmp.v17i5.6118

**Published:** 2016-09-08

**Authors:** Michalis Aristophanous, Yelin Suh, Pai C. Chi, Luke J. Whittlesey, Scott LaNeave, Mary K. Martel

**Affiliations:** ^1^ Department of Radiation Physics The University of Texas MD Anderson Cancer Center Houston TX USA

**Keywords:** ArcCHECK, IMRT, patient‐specific QA

## Abstract

Many devices designed for the purpose of performing patient‐specific IMRT/VMAT QA are commercially available. In this work we report our experience and initial clinical results with the ArcCHECK. The ArcCHECK consists of a cylindrical array of diode detectors measuring entry and exit doses. The measured result is a cumulative dose displayed as a 2D matrix. The detector array requires both an absolute dose calibration, and a calibration of the detector response, relative to each other. In addition to the calibrations suggested by the manufacturer, various tests were performed in order to assess its stability and performance prior to clinical introduction. Tests of uniformity, linearity, and repetition rate dependence of the detector response were conducted and described in this work. Following initial testing, the ArcCHECK device was introduced in the clinic for routine patient‐specific IMRT QA. The clinical results from one year of use were collected and analyzed. The gamma pass rates at the 3%/3 mm criterion were reported for 3,116 cases that included both IMRT and VMAT treatment plans delivered on 18 linear accelerators. The gamma pass rates were categorized based on the treatment site, treatment technique, type of MLCs, operator, ArcCHECK device, and LINAC model. We recorded the percent of failures at the clinically acceptable threshold of 90%. In addition, we calculated the threshold that encompasses two standard deviations (2 SD) (95%) of QAs (T95) for each category investigated. The commissioning measurements demonstrated that the device performed as expected. The uniformity of the detector response to a constant field arc delivery showed a 1% standard deviation from the mean. The variation in dose with changing repetition rate was within 1 cGy of the mean, while the measured dose showed a linear relation with delivered MUs. Our initial patient QA results showed that, at the clinically selected passing criterion, 4.5% of cases failed. On average T95 was 91%, ranging from 73% for gynecological sites to 96.5% for central nervous system sites. There are statistically significant differences in passing rates between IMRT and VMAT, high‐definition (HD) and non‐HD MLCs, and different LINAC models (p‐values <<0.001). An additional investigation into the failing QAs and a comparison with ion‐chamber measurements reveals that the differences observed in the passing rates between the different studied factors can be largely explained by the field size dependence of the device. Based on our initial experience with the ArcCHECK, our passing rates are, on average, consistent with values reported in the AAPM TG‐119. However, the significant variations between QAs that were observed based on the size of the treatment fields may need to be corrected to improve the specificity and sensitivity of the device.

PACS number(s): 87.55.Qr, 87.56.Fc

## I. INTRODUCTION

Intensity‐modulated radiation therapy (IMRT) at its introduction in the late 1990s generated tremendous excitement.^1^, ^2^, ^3^, ^4^ The anticipation was that the new technique would accurately target tumors,^5^, ^6^ while at the same time sparing adjacent normal tissues.^(7)^ IMRT achieves high levels of conformality by utilizing multiple segments from each beam with the multileaf collimators (MLCs). This can create optimal fluence maps through a process known as inverse planning.^(8)^ The fluence maps of each beam are optimized such that a set of dose constraints regarding target coverage and normal tissue sparing are satisfied. This optimization results in maximizing coverage of the target volume while minimizing dose to adjacent normal structures. Steep dose gradients between tissues are often created to achieve these goals. The nonintuitive nature of the optimization, coupled with the potential of large dosimetric errors caused by the steep dose falloff away from the target, necessitated the introduction of patient‐specific plan quality assurance (QA).^9^, ^10^ The complexity of IMRT treatments has only increased over the years, primarily by the introduction of volumetric‐arc therapy (VMAT),^(11)^ where, in addition to the MLCs, the gantry and repetition rate change during delivery as well.

The use of IMRT and VMAT is standard of care for many treatment sites. Out of 81 patients currently under treatment for head and neck cancer at our institution, 63 or 78% are being treated with IMRT (n=38 patients) or VMAT (n=25 patients), with the rest receiving 3D conformal, electron, or proton treatment. One of the most reliable methods for obtaining an absolute dose measurement in the clinic is the use of a properly calibrated ionization chamber.^(12)^ That was the first instrument used for patient‐specific QA at our institution. However, an ion chamber only offers the possibility of making a single point measurement, and this can be an inadequate verification of a treatment plan, particularly in the case of large fields with complex dose distributions. Therefore a common approach to assess delivery accuracy that was adopted early on was the use of a phantom made up of solid water slabs. This allowed both an absolute dose measurement using an ion chamber and a relative dose measurement using one or multiple radiographic films placed in between the solid water slabs.^(11)^ The disadvantage of this approach is that it involves the use of films, which not only have energy and directional dependence, but also need development and calibration to convert optical density to dose. The use of a film processor for development is complicated by the fact that it needs to be carefully maintained to achieve a reproducible development process.

Several newer devices have been made commercially available over the last decade, all of which attempt to simplify patient‐specific IMRT/VMAT QA. Newer devices include 2D detector arrays, such as the MatriXX (IBA Dosimetry, Schwarzenbruck, Germany) and MapCHECK (Sun Nuclear Corporation, Melbourne, FL), and 3D detector arrays, such as the Delta^4^ (ScandiDos, Uppsala, Sweden) and ArcCHECK (Sun Nuclear Corporation). The MatriXX is a 2D ion chamber array that can be either placed inside a solid water phantom to measure the dose distribution of a whole treatment plan in one plane or mounted on the LINAC head to measure the fluence from each beam. A similar device is the MapCHECK, which utilizes a 2D diode detector array instead. The Delta^4^, on the other hand, employs a 3D array of diode detectors on two orthogonal planes. The other 3D detector array, the ArcCHECK, is used in this study and will be discussed in detail in the next sections.

In the past five years, several studies reported experience with the ArcCHECK.^13^, ^14^, ^15^, ^16^ Yan et al.^(13)^ reported on developing a calibration procedure for the detector array to account for both the intrinsic differences between the detectors, as well as correcting for any angular dependence. Neilson et al.^(14)^ reported on their experience with the ArcCHECK device in the clinical setting, presenting passing rates from various treatment sites. They compared isocentric deliveries with the target in the center of the device and after a shift so that the high dose target fell on the diode detectors. They found that isocentric delivery with the ArcCHECK is sufficient, leading to reduced QA times and comparable results with high dose delivery. Li et al.^(15)^ presented their commissioning results where they made several measurements to investigate dose response to changing repetition rates, MUs, and field size. They found acceptable response under the tested conditions and concluded on the suitability of the ArcCHECK for IMRT/VMAT QA. Finally, Chaswal et al.^(16)^ presented a comprehensive set of commissioning measurements. They found the diode detectors to have an acceptable response to all tested conditions. They concluded that the 2%/2 mm criterion for evaluating QA exhibited higher sensitivity in evaluating VMAT plans than the 3%/3 mm criterion.

Commonly accepted IMRT QA criteria are based on the AAPM task group 119 (TG‐119) reported measurements.^(9)^ However, these measurements were taken under specific conditions and it is unclear whether they are transferrable to alternative approaches. In addition, recent studies questioned some of the findings of the report. Kruse^(17)^ reported on the accuracy of performing field‐by‐field QA for IMRT plans with two different planar dosimeters. The study found that gamma analysis scores could not reliably identify plans with low dosimetric accuracy. Kruse concluded that an effective QA should include a composite dose measurement. Nelms et al.^(18)^ developed a method to calculate 3D dose distributions in a patient geometry using the ArcCHECK measurements and the patient anatomy from the planning CT. They found that gamma passing rates in excess of 90% were possible with the more stringent 2%/2 mm criterion. Despite these reports and the fact that newer devices differ in construction, materials used, and measurement locations, the 3%/3 mm criterion as mentioned in TG‐119 is typically acceptable in the clinical setting.^(16)^ Following appropriate commissioning of our ArcCHECK devices, we introduced them clinically with the above‐mentioned criteria for passing IMRT/VMAT QAs. In this report, we briefly describe the most important commissioning measurements, and present and analyze the results with the ArcCHECK following its first full year of clinical operation, with particularly emphasis on QA failure trends.

## II. MATERIALS AND METHODS

### A. ArcCHECK device and its calibrations

The ArcCHECK is an acrylic (PMMA) cylindrical phantom with a density of $1.15\;g/{\text{cm}}^{3}$. The device has a diameter of 26.6 cm and a length of 21 cm. The central cavity has a diameter of 15 cm and can accommodate various inserts (e.g., solid homogeneous core, dosimetric core). The diode array consists of 1,386 diode detectors ($0.8\times 0.8\;{\text{mm}}^{2}$) arranged in a helix with a pitch of 1 cm. The diameter of the cylindrical diode detector array plane is 20.8 cm and the diode detectors are spaced 1 cm apart both along the cylindrical length and along the circumference.

The detector array and dose calibrations are necessary steps for making measurements using the ArcCHECK. We performed an in‐house calibration following manufacturer procedures for each beam energy utilized clinically for IMRT or VMAT plans (6, 15, and 18 MV). The first step is the detector array calibration, which measures and corrects for the relative sensitivity of the detectors.^13^, ^19^ This calibration is intended to be applied to the raw measurements of each detector to eliminate relative response differences between individual detectors. The dose calibration consists of a single 200 MU delivery with a $10\times 10\;{\text{cm}}^{2}$ field. The actual dose delivered to the diode detectors was entered in the software (SNC Patient, Sun Nuclear Corporation, Melbourne, FL) and a calibration factor was obtained. The known delivered dose to the detectors was calculated using our clinical treatment planning system (TPS) (Pinnacle^3^, Philips Healthcare, Fitchburg, WI) and the virtual phantom provided by Sun Nuclear Corporation with a density override of $1.15\;g/{\text{cm}}^{3}$. In the TPS, a point at a depth of 2.9 cm was identified (diode detector depth), and the dose for 200 MUs delivered with a $10\times 10\;{\text{cm}}^{2}$ field was calculated and recorded. The Pinnacle dose calculation conditions for this diode dose measurement were the same as for clinical patient plans (i.e., the dose grid was set to a 0.3 cm cubic size) and the collapsed cone convolution (CCC) algorithm was used for dose calculations. This diode dose obtained in the TPS was compared with a manual calculation made by assuming a water‐equivalent, flat‐phantom geometry.

### B. Basic characteristics of detector array responses

In order to verify the uniformity of the detector responses following an array calibration, we delivered three arcs with a 6 MV beam and a field size large enough to cover the entire array ($27\times 27\;{\text{cm}}^{2}$): the gantry angle from 270° to 90° with the proper ArcCHECK setup (200 MUs), and 270° to 0° and 1° to 90° with the ArcCHECK device rotated by 180° to capture the bottom part of the array without going through the couch (100 MUs each). These deliveries when combined give a uniform dose to all the detectors and the dose should agree with that for the same arcs calculated in the TPS. To match the actual delivery, the three arc plans, one from 90° to 180°, one from 181° to 270°, and one from 270° to 90°, were calculated in the TPS. In addition, the relative difference between detectors was calculated from the measured data by normalizing to the average over all detectors.

Furthermore, tests for the linearity and repetition‐rate dependence of the detector response and the response of the detector array to simple field irradiation were performed. The ArcCHECK was setup isocentrically for these measurements (i.e., with the isocenter at the center of the device).^(20)^ For the linearity, we irradiated the ArcCHECK device with a $10\times 10\;{\text{cm}}^{2}$ field and 1–600 MUs at a repetition rate of 600 MU/min. For each irradiation, the measured dose was obtained by taking a region of interest (ROI) in the central $5\times 5\;{\text{cm}}^{2}$ region of the irradiated field. All the deliveries were also calculated using a $10\times 10\;{\text{cm}}^{2}$ field in the TPS. For the repetition rate dependence, 100 MU was delivered with a $10\times 10\;{\text{cm}}^{2}$ field and the repetition rate varying from 100 to 600 MU/min in 100 MU increments. The dose was measured with a $3\times 3\;{\text{cm}}^{2}$ ROI placed in the center of the irradiated field and all the deliveries were again calculated in the TPS. Finally, four simple static fields were delivered:^(16)^ $5\times 5\;{\text{cm}}^{2},5\times 5\;{\text{cm}}^{2}$ with a 10 cm lateral shift, $10\times 10\;{\text{cm}}^{2}$, and $25\times 27\;{\text{cm}}^{2}$.^(19)^ The measured dose was compared with the calculated in the TPS.

### C. Comparison with previous QA system

Prior to the introduction of the ArcCHECK in our clinic, the I'mRT solid water phantom (IBA Dosimetry, Schwarzenbruck, Germany) with a film and an ion chamber was the standard QA procedure for IMRT/VMAT at our institution. For 31 patients whose QAs were performed with the phantom, QAs were repeated with the ArcCHECK device. The QA procedure followed for the ArcCHECK was the same as the one for the film and ion chamber. The patient plan was mapped on a CT scan of the ArcCHECK device, imaged with the uniform plug, and the DICOM dose and plan data were obtained. The QA results from the ArcCHECK were compared with those with the film and ion chamber. The ion chamber was used for an absolute dose comparison (3% criterion) and the film for a relative comparison (90% threshold at 5%/3 mm). The plans that were selected for this comparison included 26 IMRT and 5 VMAT patients for different treatment sites: five in thoracic, seven in head and neck, two gynecological, three gastrointestinal, two hematology, four genitourinary, five central nervous system, one sarcoma, and two pediatric.

### D. ArcCHECK patient‐specific QA protocol

Based on the calibration, test results, and the AAPM TG‐119 report, we developed a protocol for our clinical patient‐specific IMRT/VMAT QA. Based on this protocol, the dose of every IMRT or VMAT patient plan is recalculated on the ArcCHECK virtual phantom as recommended by the manufacturer. Typically, the plan isocenter is placed at the center of the cylindrical phantom; however, in certain cases a shift might be needed (typically, in the superior or inferior direction only) to avoid irradiating the electronics or to fit the irradiated field onto the device. The dose calibration is performed before any QA session with the nominal dose used for calibration, as was the practice for our previous film and ion chamber system. The array calibration is performed every six months at our institution, to account for unequal changes in the diode response caused by radiation damage. Following delivery, the measured dose is compared using the SNC Patient software to the planned dose exported from the TPS. The results are evaluated using gamma analysis with a threshold of 10%. A QA passes if greater than 90% of points meet the 3%/3 mm criterion. For evaluation, the measurement uncertainty and the Van Dyk percent difference^(21)^ are enabled. The measurement uncertainty is a correction applied within the software after it makes an estimate of the daily uncertainty. The Van Dyk method calculates the percent difference normalized to the global maximum dose difference in the plan as opposed to a local maximum.

### E. Patient‐specific QA

**Figure 1:**
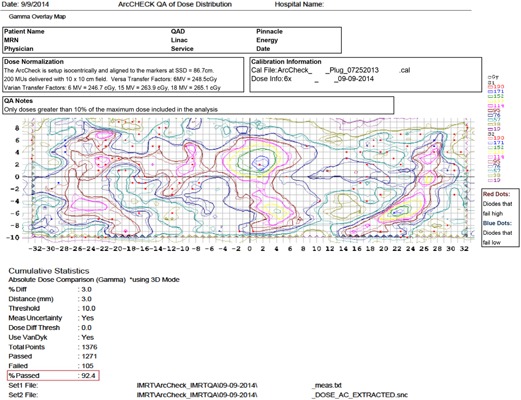
An example QA report at our institution.

Three thousand one hundred and sixteen treated cases were QAed on two ArcCHECK devices at our institution between October 2013 and September 2014. Treatment planning for all patients was performed using Pinnacle^3^ v.9 (Philips Healthcare, Fitchburg, WI). The dose grid is typically set so that it covers at least the 20% isodose line and the grid resolution is 0.3 cm in all directions. Treatment planning guidelines vary by clinical service group; however, there are certain parameters that are common across different treatment sites. For IMRT, a step‐and‐shoot technique is used with five to nine beam angles, most commonly chosen along with the Direct Machine Parameter Optimization (DMPO) algorithm for optimization. The beams are split if the width of the field (X direction for Varian LINACs) is greater than 20 cm. The total number of control points (CPs) is usually set to 15 per beam. The minimum segment area is set to 4 cm and the minimum number of MUs to 2 per segment. For VMAT, SmartArc optimization is utilized, and typical guidelines include using 2 to 3 arcs with the leaf speed limit set to 0.46 cm per degree and the gantry spacing set to 3° or 4°. For both IMRT and VMAT, the jaws are fixed during beam delivery. For all patients, the standard clinical QA protocol as described previously was followed. All QA results are for individual patients, not individual fields. An example of the main page of a QA report at our institution is shown in Fig. 1.

The report also includes the patient identifying information, treatment site, clinical service group, beam energy, treatment technique, LINAC model, LINAC, serial number of the device, and person who performed QA. The results were compared using the gamma passing rate (“%Passed” in the report) by grouping them into different categories. The categories used to sort the data were clinical service group, treatment technique, type of MLCs, LINAC model, device, and operator. To evaluate differences between categories, we tested the different groups using a two‐tailed t‐test evaluated at a 0.05 level of significance. Assuming that a good threshold for QA, defined as one that flags potentially problematic plans, is one that flags one in twenty QAs, we recorded the gamma passing rate threshold that would result in 95% of QAs to pass (T95) for each category.

We performed additional analysis for the failed QA cases in order to further investigate the reason for the failures. We performed two separate comparisons with the ion chamber data for a subset of the QAs. As part of an initial investigation into the failing cases, over the course of one month, all the QAs performed in a single QA session every night were delivered with the ion chamber placed in the central insert. This resulted in 81 QAs evaluated with the ArcCHECK also having a point ion chamber measurement at the center of the device. This investigation included both passing and failing cases. For further analysis, 31 QAs that failed with the ArcCHECK were repeated with our previous film and ion chamber system. Finally, certain plan characteristics of all the failed cases with the ArcCHECK were recorded. We looked at the field size (X and Y jaws), total number of MUs, and MUs/CP. In order to better understand the nature of these failures, we selected the equivalent number of the best passing QAs and recorded their plan characteristics for comparison.

## III. RESULTS

### A. Calibrations and initial tests

Assuming a water‐equivalent flat phantom geometry, the depth of the diode detectors is equivalent to a depth of 3.3 cm in water, and the ArcCHECK is set up isocentrically with a source‐to‐surface distance (SSD) of 86.3 cm. Our LINACs are calibrated at the reference depth of the maximum dose ($d_{\text{max}}$) with the reference SSD (${\text{SSD}}_{0}$) of 100 cm to deliver 1 cGy per MU. Therefore the source‐to‐calibration distance (SCD) is ${\text{SSD}}_{0}+d_{\text{max}}$. Table 1 compares the manually calculated detector dose ($D_{\text{man}}$) in the water‐equivalent geometry and the dose to the point at the detector depth inside the cylindrical virtual phantom calculated in the TPS ($D_{\text{TPS}}$) for 6 MV, 15 MV, and 18 MV, respectively. The difference between $D_{\text{man}}$ and $D_{\text{TPS}}$ was within 0.5% for all energies.

**Figure 2:**
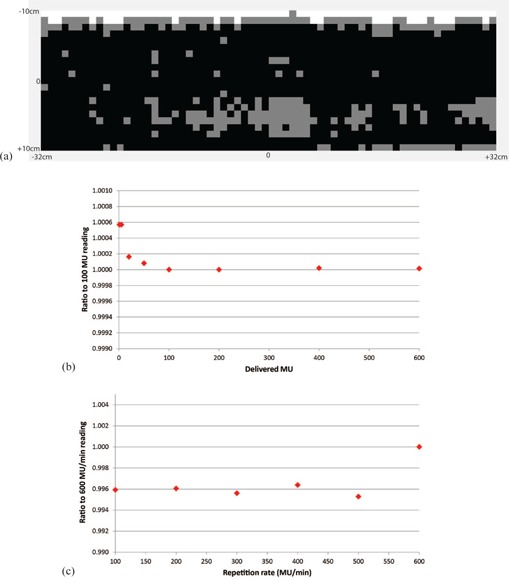
Uniformity (a), linearity (b), and repetition rate dependence (c) of the detector response. Uniformity map: difference >2% (white), 1%< difference <2% (gray), and difference <1% (black).

For the detector response uniformity verification, the average detector difference between the composite measurement and the plan calculated in the TPS was found to be 1.3% with a standard deviation of 1%. For the point by point detector comparison from the mean dose over all detectors, we found a variation of 1% (1 SD) with all the points with greater than 2% variation at the edge of the device (away from the electronics) as shown in Fig. 2(a). Figure 2(b) shows a plot of the dose measured with the ArcCHECK normalized to the 100 MU reading against the delivered MUs, illustrating the linearity of the detector response. In addition, when comparing the measured dose with the TPS calculated dose, the difference is within 1% for all

**Table 1:** Manual ($D_{\text{man}}$) and TPS ($D_{\text{TPS}}$) calculation of the dose to the detectors to be used for the dose calibration.

*Energy (MV)*	$D_{\text{man}}(\text{cGy})$	$D_{\text{TPS}}(\text{cGy})$	*Difference (%)*
6	247.0	246.7	0.1
15	263.8	263.9	−0.04
18	265.8	265.1	0.3

MU deliveries. Figure 2(c) shows a plot of the dose measured with the ArcCHECK normalized to the 600 MU/min reading against the repetition rate, illustrating the repetition rate dependence of the detector response. Again, the difference between the dose measured with the ArcCHECK and the dose calculated in the TPS was less than 1% for all repetition rates. The average dose over all repetition rates was 123.1 cGy with a standard deviation of 0.2 cGy.

Simple static field deliveries to verify the dose calibration for the entire detector array showed greater than 95% gamma passing rate for all fields, when compared with the TPS calculated dose, at the 2%/2 mm criterion. The results of the comparison of our previous QA system with the ArcCHECK system for both the ion chamber and gamma passing rate are summarized in Table 2. The gamma for this comparison was evaluated at 5%/3 mm since it was the criterion used for film with our previous clinical patient specific QA procedure.

**Table 2:** The average (± SD) ion chamber measurement difference and gamma passing rate, Γ, for the ArcCHECK and *I'mRT* phantom.

*ArcCHECK*		*I'm RT Phantom*
*Average Ion Chamber (%)*		
−0.1±1.7		‐0.45±1.3
	*Average* Γ (3%/3 mm) (%)	
*ArcCHECK*		I'm RT *Phantom*
98.95±1.1		98.9±1.4

### B. Patient‐specific QA

**Figure 3:**
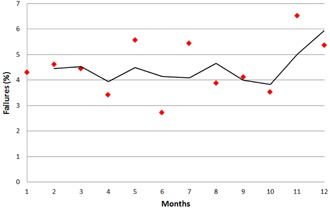
A plot of the percentage of failures per month, along with a fitted moving average trend line.

**Figure 4:**
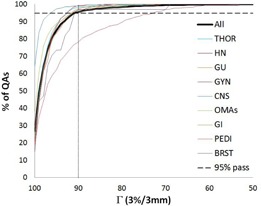
The cumulative percentage of the distributions of the gamma passing rate, Γ, (3%/3 mm) for each clinical service group individually and all together. THOR = thoracic; HN = head and neck; GU = genitourinary; GYN = gynecological; CNS = central nervous system; OMAs = lymphoma, melanoma, sarcoma; GI = gastrointestinal; PEDI = pediatric; BRST = breast.

A summary of the results from one year of clinical operation of the ArcCHECK are shown in Table 3 arranged by clinical service group. Overall, we observed a failure rate of 4.5% (140/3116) at the 90% gamma analysis threshold. A plot of the failure rate by month is shown in Fig. 3. The highest number of failures was seen in the gynecological (GYN) group with 22% of the cases failing, while the lowest failure rate was seen in the central nervous system (CNS) group with less than 1% of the cases failing. On average the threshold necessary for 95% of QAs to pass (T95) was 91%, ranging from 73% for the GYN group to 96.5% for the CNS. Figure 4 shows a histogram plot of the percentage of QAs against the gamma passing rate to illustrate the differences between the service groups.

**Table 3:** The patient QA breakdown by clinical service group, showing the number of failed QAs.

	*THOR*	*HN*	*GU*	*GYN*	*CNS*	*OMAs*	*GI*	*PEDI*	*BRST*	*Total*
# of QAs	780	687	366	363	316	243	231	96	34	3116
# of QAs for Γ<90%	16	7	11	80	3	10	8	3	2 140
% failures	2.1%	1.0%	3.0%	22.0%	0.9%	4.1%	3.5%	3.1%	5.9% 4.5%
T95	92.5%	92.5%	91.5%	73.0%	96.5%	91.5%	91.0%	91.5%	90.8% 91.0%

THOR = thoracic; HN = head and neck; GU = genitourinary; GYN = gynecological; CNS = central nervous system; OMAs = lymphoma, melanoma, sarcoma; GI = gastrointestinal; PEDI = pediatric; BRST = breast; Γ =gamma passing rate (3%/3 mm); T95 = threshold for 95% of QAs to pass.

**Figure 5:**
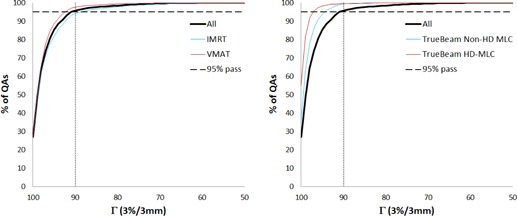
The cumulative percentage of the distributions of the gamma passing rate, (Γ,3%/3 mm) for (left) different treatment technique and (right) MLC types. HD = high‐definition.

Table 4 shows the number of QAs by treatment technique (IMRT vs. VMAT), type of MLCs in TrueBeam LINACs (Varian Medical Systems, Palo Alto, CA) (high‐definition [HD] vs. non‐HD), LINAC model (TrueBeam LINACs vs. Varian 2100 LINACs [Varian Clinac 2100, Varian Medical Systems]), device (#1 vs. #2), and operator (#1 vs. #2). While the number of QAs for IMRT and VMAT were similar, twice as many failures were observed with IMRT. For the TrueBeam LINACs, the failure rate of both HD and non‐HD was less than 1%. The TrueBeam LINAC model had a failure rate less than 1%, while the Varian 2100 LINAC model had a failure rate close to 6%. There was a statistically significant difference between techniques, MLC types, and LINAC models, but no statistically significant difference between devices or operators. T95 for the technique and MLC type are illustrated in Fig. 5, using a histogram plot of percentage of QAs against the gamma passing rate. For IMRT, T95 was 88.5% compared to 92.4% for VMAT, while T95 for HD MLCs against non‐HD MLCs was much higher, with the non‐HD being 94.5% and the HD 97.1%.

As part of our initial investigation into the failures, for 81 QAs the ion chamber was inserted in the provided ArcCHECK central cavity. Out of the 81 cases 10 QAs failed with the ion chamber and 13 with the ArcCHECK. Taking the ion chamber measurement as ground truth, we calculated the sensitivity of the ArcCHECK in detecting QA failures as 50%, with a specificity of 89%. One problem with these measurements, however, was that the QA was optimized for an ArcCHECK measurement and the ion chamber was placed in the ArcCHECK at the central location without consideration of the location of the chamber. In order to better understand the performance of the device, 31 failed QAs with the ArcCHECK were repeated with our old system. Out of the 31 cases, 10 QAs had a failed ion chamber measurement. We did not include passing QAs in this analysis, therefore we could not calculate sensitivity and specificity.

**Table 4:** The patient QA breakdown by technique, MLC type, LINAC model, device, and operator. Note that MLC type comparison is for the QAs delivered in the TrueBeam LINACs only (1,136 in total), LINAC model comparison excludes the plans calculated in the TPS with the Elekta Versa LINAC model (3,082 in total), and operator comparison excludes the operators who performed the lower number of QAs (2,796 in total).

	*Technique*		*MLC‐type*		*LINAC Model*		*Device*		*Operator*	
	*IMRT*	*VMAT*	*HD*	*Non‐HD*	*TrueBea*	*m 2100*	*#1*	*#2*	*#1*	*#2*
# of QAs	1601	1515	492	644	809	2273	1552	1564	1382	1414
# of QAs for Γ<90%	99	41	3	6	6	134	65	75	54	61
% failures	6.2%	2.7%	0.6%	0.9%	0.7%	5.9%	4.2%	4.8%	3.9%	4.3%
p‐value	<<0.001		<<0.001		<<0.001		0.39		0.64	

HD = high‐definition; 2100 = Varian Clinac 2100; Γ=gamma passing rate (3%/3 mm).

**Figure 6:**
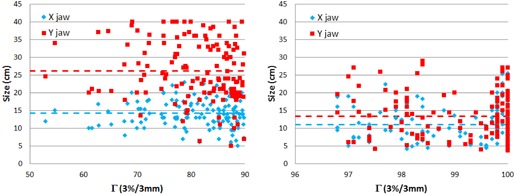
The × and Y jaw size plotted against the gamma passing rate, Γ, (3%/3 mm) separately for the failed QAs (left) and the passing QAs (right).

In the next step of the failure investigation, we took the 140 failed cases and matched them by clinical service group to the same number of cases with the best passing rates. For both the passing and the failing QAs we recorded the average × jaw size, maximum Y jaw size, total number of MUs, and total number of CPs from which we calculated MUs/CP. The Pearson correlation coefficient between the passing rate and each one of these parameters studied is shown in Table 5. While there were differences between the failed and passing cases for all of the parameters studied, the gamma passing rate is most strongly correlated with the size of the Y jaw (indicative of a larger field size). Figure 6 shows a plot of the × and Y jaw size for all the failed cases (left) and all the selected best cases (right) against the gamma passing rate. The mean Y jaw size for the failed cases is 26.2 cm, while the mean Y jaw size for the passing cases is approximately half, 13.5 cm. The difference between the mean jaw size of the two groups is statistically significant (two‐tailed t‐test, p‐value <<0.001).

**Table 5:** Correlation coefficients between the passing rates for the failed and matched passing QAs with the × and Y jaw sizes, the total number of MUs, and the MUs per control points (CPs). All correlations are statistically significant (p‐value <0.05).

	*Γ*	*X Jaw Size*	*Y Jaw Size*	*Total MU*	*MUs/CP*
Γ	1.00	−0.28	−0.62	−0.18	0.12
X jaw size	−0.28	1.00	0.72	0.12	−0.40
Y jaw size	−0.62	0.72	1.00	0.20	−0.30
Total MU	−0.18	0.12	0.20	1.00	0.01
MUs/CP	0.12	−0.40	−0.30	0.01	1.00

*Γ* = gamma passing rate (3%/3 mm).

## IV. DISCUSSION

Both ArcCHECK devices currently in clinical operation at our institution were tested and characterized before being introduced into the clinic for patient‐specific IMRT/VMAT QA. In this work we presented the most representative set of tests that were performed as part of the commissioning process, the collection and analysis of the results from the first full year of operation of both devices in our clinic, as well as our initial investigation into the failures. Our results show the observed trends in the passing rates and appropriateness of the clinically selected passing threshold.

The dose calibration is one of two tasks that need to be performed before making any QA‐related measurements. Performing the dose calibration with the ArcCHECK is complicated by the fact that it is difficult to take equivalent ion chamber dose measurements due to the cylindrical geometry. However, it is possible to make an ion chamber measurement using the flat‐water phantom approximation. In this work we chose to perform two independent calculations of the detector dose: a TPS dose calculation was confirmed with a manual calculation for the water‐equivalent flat phantom geometry. The agreement in the dose calibration was less than 0.5% for all energies. Using the dose calibration factor calculated in the TPS seems the most appropriate since, due to the cylindrical geometry of the ArcCHECK, both the manual calculation and an actual ion chamber measurement would be made in an approximate geometry.

By making the arc irradiation measurements (different angles) we can verify the array calibration, since the entire array “sees” both entry and exit doses. For the posterior gantry angles, we rotated the ArcCHECK by 180° to make sure that the beam did not pass through the couch, which we found to have a significant adverse effect on the passing rate and to introduce additional uncertainty in the measurement. The detector array uniformity was largely within 2%, except at the superior edge where the measured dose was lower than average by as much as 3.4%, as shown in Fig. 2(a). This difference may be attributed to those detectors being closer to the device edge and therefore having reduced scatter contribution. In addition, we demonstrated the linearity of the detector response, as shown in Fig. 2(b) where the differences between different delivered MUs are less than 0.5%. The results also indicate that the detector response is practically independent of the repetition rate, with the measured dose differences less than 0.6%, as in Fig. 2(c). This is within the repetition rate dependence reported by the manufacturer, which is ±1% for SSDs ranging from 75 to 250 cm. Finally, by monitoring the failure rate on a monthly basis we determined that there was a slight increase in the percent of failures closer to the end of the one year (greater than 5% failure rate for two months in a row), as shown in Fig. 3. To avoid any bias introduced in the results by radiation damage to the detectors, we determined that we need to recalibrate the device every six months (or approximately 1,600 QAs).

A comparison with the gold standard is an important verification step when transitioning from one QA procedure to another to ensure the consistency in the results. Therefore, several patient QAs were performed with both the ArcCHECK and our previous I'mRT phantom with the film and ion chamber. The results of the patient‐specific QA evaluation (i.e., passing rates) showed no difference between the two systems (Table 2). However, this initial investigation included only passing QAs. In the efforts to troubleshoot failing QAs we performed additional comparisons with ion chamber measurements, where we included cases that failed with the ArcCHECK. First, we compared the results of 81 QAs with the ArcCHECK and the ion chamber embedded in the device. These results showed good specificity; however, somewhat reduced sensitivity. Given the fact that these QAs were optimized for ArcCHECK delivery (i.e., shifted only in the superior–inferior direction without making an effort to put the ion chamber in a flat dose region), large uncertainty in the ion chamber readings could be expected. Therefore, we performed a second analysis where for 31 QAs that failed with the ArcCHECK we repeated the QA with our previous QA system where the location of the ion chamber was optimized. Those measurements showed that only 10 (or 32%) QAs failed the ion chamber measurement and therefore agreed with the ArcCHECK. However, we did not include passing cases in that analysis, and therefore we could not reliably calculate sensitivity and specificity. While both approaches have limitations, they do offer important information about the ArcCHECK performance when compared to a gold‐standard detector. Specifically, the ArcCHECK seems to be able to identify cases that fail with our gold standard (high sensitivity), however, tends to flag as failed many passing cases (low specificity).

The initial clinical results with the ArcCHECK also reveal some interesting trends. The average gamma passing rate over all patients in order for 95% of QAs to pass is 91%, which is close to our clinically chosen threshold of 90%. These values also agree well with the reported threshold of 88%–90% for composite plans from the AAPM TG‐119. It is interesting to note the two thresholds agreeing, given that they result from different QA device designs (2D vs. 3D). However, despite the on‐average agreement, significant variations between clinical service groups, treatment techniques, MLC types, and LINAC models were observed. From clinical experience, one reason that some QAs fail more than others with the ArcCHECK seems to be the size of the treated region. Specifically, larger field sizes, particularly in the longitudinal direction, were observed to fail more often than the rest.

In order to investigate this further, we matched the number of failed QAs to passing cases (picking the same number of cases proportionally from each clinical service group), by selecting the QAs with the highest passing rates from each service group. We found that the passing rate has the largest correlation with the Y jaw size (Table 5), indicating that a larger Y jaw size results in lower gamma passing rates. In order to look at this more closely, we plotted in Fig. 6 the size of the × and Y jaws against the gamma passing rate for the failed and passing QAs separately. A clear difference is seen between the size of the Y jaw in the two instances. For the failed cases the mean is around 26 cm, while for the passing it is around 14 cm and closer to a square field (more similar to × jaw size). This result is also supported by the fact that the clinical service group with the higher number of failures, GYN, frequently treats the pelvis and paraaortic nodes, which result in long fields (< 30 cm) in the longitudinal direction. In addition, IMRT treatments with higher failures see larger field sizes than VMAT, HD MLCs with higher passing rates are limited to a field size of 22 cm, and LINAC model shows lower average passing rate for Varian 2100 LINAC, which is where all the GYN cases are treated. In fact, if the GYN cases are all left out of the failures, this leaves 48 failed cases for all other service groups and they are almost exactly divided to 50% IMRT and 50% VMAT, not supporting that the ArcCHECK favors one intensity‐modulation technique over the other, but rather reinforcing the field size dependence of the device.

Feygelman et al.^(22)^ first observed a field size dependence with the ArcCHECK after delivering single static fields with and without the homogeneous core seeing a change from −1.1% to 1.3% and −0.7% to 1.7%, respectively, when going from a $5\times 5\;{\text{cm}}^{2}$ to a $25\times 25\;{\text{cm}}^{2}$ field size. This field size dependence was confirmed by Li et al.,^(15)^ who reported a 1.7% increase in sensitivity of the detectors for a $20\times 20\;{\text{cm}}^{2}$ field size. Our results show that the field size, particularly as defined in the longitudinal direction, where it can be as large as 40 cm, has a measurable effect on passing rates of clinical QAs as well. Possible strategies to mitigate the effects of this field size dependence on clinical QAs could include: 1) the application of correction factors for this field size dependence as done by Feygelman and colleagues, 2) performing a dose calibration with a field size that more closely matches the delivered QA, or 3) including the field size dependence in the gamma analysis, for example at 4%/3 mm instead of 3%/3 mm if this dependence is measured as 1%.

## V. CONCLUSIONS

This work describes some of the procedures followed in commissioning the ArcCHECK device for patient‐specific IMRT/VMAT QA and the results of its initial implementation in our clinic. Commissioning resulted in characterizing the output and response of the device and revealed that it performs as expected. Initial clinical results are mixed. They indicate that, on average, the passing threshold used clinically is comparable to the AAPM TG‐119 report (i.e., 90% at 3%/3 mm). However, our clinical results show a field size dependence in the passing rates. This means that, if our goal is to investigate the lowest‐performing 5% of QAs, variations based on field size may result in too many unwarranted QA failures (e.g., larger field sizes) or nearly all the QAs passing (e.g., CNS cases). This field size dependence should be taken into account when evaluating QAs with the ArcCHECK in order to improve its specificity for large field size plans; however, care has to be taken to not decrease its sensitivity in detecting failures while correcting for this dependence.

## COPYRIGHT

This work is licensed under a Creative Commons Attribution 3.0 Unported License.
